# A digital intake approach in specialized mental health care: study protocol of a cluster randomised controlled trial

**DOI:** 10.1186/s12888-017-1247-9

**Published:** 2017-03-07

**Authors:** Margot J. Metz, Iman Elfeddali, David G. H. Krol, Marjolein A. Veerbeek, Edwin de Beurs, Aartjan T. F. Beekman, Christina M. van der Feltz-Cornelis

**Affiliations:** 1 EMGO Institute for Health and Care Research (EMGO+), VU University, Amsterdam, The Netherlands; 2 GGz Breburg, Mental Health Institute, Postbus 770, 5000 AT, Tilburg, The Netherlands; 3 School for Public Health and Primary Care (CAPHRI), Maastricht University, Maastricht, Netherlands; 4 TRANZO Department, Tilburg University, Tilburg, The Netherlands; 5 Netherlands Institute of Mental Health and Addiction (Trimbos Institute), P.O. Box 725, 3500 AS, Utrecht, The Netherlands; 6 Department of Clinical Psychology, University of Leiden, Leiden, The Netherlands; 7 Foundation Benchmark Mental Health Care, Stichting Benchmark GGZ, Rembrandtlaan 46, 3723 BK Bilthoven, The Netherlands; 8 Department of Psychiatry, VU University Medical Centre, Amsterdam, The Netherlands; 9 GGZ inGeest, Mental Health Institute, A.J. Ernststraat 1187, 1081 HL Amsterdam, The Netherlands

**Keywords:** Patient participation, Patient preference, Adherence to treatment, eHealth, Routine Outcome Monitoring, Shared Decision Making, Decisional conflict, Peer support, Intake, Cluster randomised controlled trial, Mental health care, Depressive disorder, Anxiety disorder, Personality disorder

## Abstract

**Background:**

Enhancing patient participation is becoming increasingly important in mental health care as patients use to have a dependent, inactive role and nonadherence to treatment is a regular problem. Research shows promising results of initiatives stimulating patient participation in partnership with their clinicians. However, few initiatives targeting both patients’ and clinicians’ behaviour have been evaluated in randomised trials (RCT). Therefore, in GGz Breburg, a specialized mental health institution, a digital intake approach was developed aimed at exploring treatment needs, expectations and preferences of patients intended to prepare patients for the intake consultations. Subsequently, patients and clinicians discuss this information during intake consultations and make shared decisions about options in treatment. The aim of this trial is to test the efficacy of this new digital intake approach facilitated by Routine Outcome Monitoring (ROM), peer support and training of clinicians as compared to the intake as usual. The primary outcome is decisional conflict about choices in treatment. Secondary outcomes focus on patient participation, shared decision making, working alliance, adherence to treatment and clinical outcomes.

**Methods:**

This article presents the study protocol of a cluster-randomised controlled trial in four outpatient departments for adults with depression, anxiety and personality disorders, working in two different regions. Randomisation is done between two similar intake-teams within each department. In the four intervention teams the new intake approach is implemented. The four control teams apply the intake as usual and will implement the new approach after the completion of the study. In total 176 patients are projected to participate in the study. Data collection will be at baseline, and at two weeks and two months after the intake.

**Discussion:**

This study will potentially demonstrate the efficacy of the new digital intake approach in mental health care in terms of the primary outcome the degree of decisional conflict about choices in treatment. The findings of this study may contribute to the roll out of such eHealth initiatives fostering patient involvement in decision making about their treatment.

**Trial registration:**

Trial registration: Dutch Trial Register NTR5677. Registered 17th January 2016.

## Background

The enhancement of patient participation in health care is deemed to be of great importance [1–3]. Research has pointed out that patients who take an active role in their treatment are often more satisfied and feel more in control with the health care they receive, are more able to manage their own mental health, have better treatment adherence, report a higher quality of life, and show better health outcomes [4–10].

Despite the importance of patient participation, the literature offers no univocal definition of this concept [10, 11]. In this study we choose the most commonly used definition of patient participation, referring to patient involvement in decision making about their health care [10, 12]. This way, patient participation, which includes behaviours such as providing information, asking questions and preparing for consultations, is an issue closely related to Shared Decision Making (SDM) [13]. SDM is defined as a collaborative approach in which clinicians and patients share the best available information when making clinical decisions, and where patients are supported to consider options to achieve informed preferences [14]. In Dutch mental health care, the importance of patient participation is emphasized by a range of initiatives aimed at stimulating Shared Decision Making [15–18]. There is growing evidence of the benefits of SDM in mental health care. Research has shown that SDM in mental health care leads to better informed and more actively engaged patients, more patient satisfaction, less decisional conflict and better patient treatment adherence [19–29].

In spite of the interest and the potential of SDM approaches in mental health care, to date many patients still have a dependent, inactive role in mental health treatment, believing ‘clinicians know best’ and expecting clinicians to make decisions in treatment [15, 16, 28–31]. Fostering patient engagement and SDM in mental health care is, therefore, essential. More initiatives are needed to support the decisional capacity, participation and active behaviour of patients in treatment granting them more choice and control regarding their own mental health care, to provide a more equitable and collaborative working relationship and to enhance shared decision making [1, 4, 13, 32].

Previous studies have shown that both the attitudes of clinicians and patients influence patient participation and joint decision making [8, 13, 30, 33–35]. The power imbalance between patients and clinicians is deemed to be a key barrier in making shared decisions [36]. Few initiatives targeting both patients’ and clinicians’ behaviour have been conducted and evaluated [33]. This underlines the need for new initiatives that aim to support patients as well as to modify clinicians’ attitudes [13, 35, 36].

It should be noted, however, that not all patients are equally capable to participate in their treatment process. Adequately fulfilling this role depends on interests, skills and support that patients receive [4, 5]. Furthermore, the patient-clinician working alliance has a great influence on the degree of patient participation in decision making [37, 38]. The working alliance between patient and clinician refers to the collaborative relationship between patient and clinicians and includes communication style, the quality of the relationship, agreement on goals and tasks and mutual decision making [37–41]. The patient-clinician relationship is an important basis for sharing treatment decisions [37, 38]. Several studies have demonstrated the positive correlation between the quality of the patient-clinician alliance with treatment adherence and clinical outcomes [9, 40, 42, 43].

### Rationale

As described above, a reversal in the current practice, focused on attitudinal changes at the level of patients as well as clinicians from the start of treatment on, is needed to mobilise and facilitate patients to participate in mental health treatment. Therefore, GGz Breburg, a specialized mental health institute in the southern part of the Netherlands, will implement a digital intake approach incorporating eHealth interventions integrated with Routine Outcome Monitoring, which implies regular measurements of clinical outcomes during treatment (for details see page 7), and facilitated by consultation of peers and clinicians’ training. It aims to explore treatment needs, expectations and preferences of patients and discussing this information with the clinicians during intake consultations. This digital intake approach is expected to influence both sides of the therapeutic dyad in a positive way and will be implemented in four outpatient departments, for adults with depression, anxiety and personality disorders. Such a combined initiative in the intake process has not been tested in a randomised controlled trial.

### Aim and hypotheses

Aim of this trial is to test the efficacy of a digital exploration of patients’ treatment preferences at intake facilitated by Routine Outcome Monitoring (ROM), peer support and training of clinicians in terms of the primary outcome the degree of decisional conflict, as an important result of patient participation and shared decision making [26, 44]. This manuscript will report on the development of this new digital intake approach and the design of the study that will be conducted to evaluate this approach.

The first hypothesis is that, compared to the intake as usual, the new intake method will lead to less decisional conflict about choices in treatment. The second hypothesis is that the implementation of the new intake method enhances patient participation, stimulates the process of shared decision making, improves the working alliance between patients and clinicians, and finally has positive effects on the adherence to treatment and clinical outcomes.

## Methods/Design

### Trial design

This study is designed as a two-arm matched-pair cluster-randomised controlled trial with two conditions: 1. A digital intake process with eHealth interventions integrated with two consecutive Routine Outcome Monitoring (ROM) measurements and consultation of peers supporting patients to participate from the beginning of treatment along with training and coaching of clinicians to adopt the changing roles; 2. The intake as usual with a single ROM measurement and without eHealth interventions, peer support and training of clinicians. The control teams will implement the new intake process after the completion of the data-collection.

### Participants and setting

**Fig. 1:**
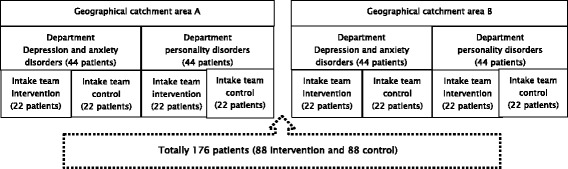
Participating departments and intake teams

This study will be conducted in four outpatient departments of a specialised mental health institution GGz Breburg, located in the southern part of the Netherlands. Annually, more than 14.000 patients are treated by about 2000 employees at GGz Breburg. The digital intake approach will be being tested among various diagnostic groups (patients with depression, anxiety and personality disorders as their primary diagnosis). Two of the participating departments are specialised in treating patients with depression and anxiety disorders; each operating in a separate geographical catchment area. The other two departments are specialised in personality disorders and are active in separate catchment areas. The four participating departments are each divided into two teams of clinicians who perform intakes (Fig. 1). Each team of intake-clinicians have their own multidisciplinary team consultation, in which treatment policy at the patient level is discussed and checked. Due to the matched pair design at the level of intake-teams within each department, in total four intervention and four control teams participate. In accordance to the sample size calculation (see section sample size, page 18) 176 patients will be included (88 in the intervention and 88 in the control teams), with an average of 44 patients per department (22 intervention and 22 control).

#### Inclusion and exclusion

Patients from the participating departments will be invited for participation in this study if a) a full intake is planned and b) when they are fluent in Dutch. Patients who meet these criteria will be asked for written informed consent. Patients will be excluded form the study if they do not receive a full intake and do not have adequate Dutch language skills (verbal and written).

### Randomisation and blinding

A cluster randomised design at the level of intake-teams and participation of intake-teams in separate multidisciplinary consultations will serve to limit contamination effects between intervention and control groups [45]. The pairs of intake groups within a department are randomly assigned to either the experimental or control condition (matched pairs). The matched teams within each department perform intakes for a similar population of patients in a similar geographic catchment area and have similar personnel. Randomisation of the intake-teams is conducted by an independent researcher, using a syntax in SPSS, which allocates the matched pairs to the control (code 0) or intervention group (code 1). To reduce bias, data collection for this study will be carried out by research assistants, independent of the principal investigator and treatment, with self-report instruments completed by both patients and clinicians. This results are not visible at patient level during intake and treatment. Only the outcome parameters no-show, drop-out and clinical outcome (ROM) will be collected in the context of treatment. As is common practice in cluster randomised RCTs, propensity analyses will be performed at the analytical stage to correct for possible biases in the randomisation process [45].

During inclusion, while informing patients and asking them for written informed consent, the research assistants are blinded for the study arm. The questionnaires are based on self report and hence do not require assessment by a research assistant. Due to the randomisation at the team level and the nature of the intervention, blinding of the clinicians and patients is impossible.

### Intervention and control

The intervention teams implement an intake intervention consisting of four parts: eHealth modules, Routine Outcome Monitoring, peer support and training of clinicians.

Firstly, eHealth modules are an accessible way for patients to prepare the face to face consultations together with relatives, offer opportunities for patients to participate actively in treatment and support shared decision making [46–48]. Secondly, Routine Outcome Monitoring (ROM) is integral part of the digital intake approach. ROM implies regular measurement of clinical outcomes, at the beginning, during and at the end of treatment, aiming to provide feedback (e.g. with graphics) on the patients’ progress during treatment and use the information to adjust treatment [49–52]. At the intake (and later on during treatment), ROM is a useful tool to involve patients in their treatment process by means of providing information by which shared decisions are made about treatment [18, 53]. In this initiative, ROM results visualised in a graphic environment, are presented as ‘the recovery trajectory of the patient’. The participating departments all use the same ROM questionnaire which measures symptoms and functioning. Two ROM measurements in the intake process may contribute to meaningful results which are not dependent on a single measurement point. Thirdly, the availability of peers for counselling is a promising approach empowering patients to participate in treatment [54]. Finally, training of clinicians and follow-up training sessions take place to stimulate and facilitate the requested changing roles of clinicians and patients [36] in the new intake approach.

The eHealth modules are designed to explore treatment needs, expectations and preferences of patients. With these modules patients prepare themselves, along with relatives, for the face to face intake consultations, which are intended to lower decisional conflict, stimulate patient participation and facilitate the dialogue between patients and clinicians about choices in treatment. The content of the eHealth modules is described in Table 1.

**Table 1:** Content of intake eHealth modules

First module a pre-intake intervention (before the first intake consultation):
- Patients are informed about psychiatric symptoms and the influence on daily life (e.g. with animations). - Patients work on questions about their life events, symptoms, the burden and impact of the mental health problems on daily life (before and after the symptoms) and the urgency which problem changing first. Preferably, this preparation takes place together with a relative. - Routine Outcome Monitoring (ROM) is integral part of the eHealth module. - Patients and clinicians, who perform intakes, get a summary of the degree of (dis)satisfaction about life domains, burden of symptoms and the recovery line (ROM). - During participation in the eHealth module, patients get the opportunity to contact with peers (by phone or mail) who can support and prepare them for the intake consult.
Second module between first and second intake consultation:
- Prior to the second face to face consultation, patients look back on the results of the first module and face to face intake consultation. The summary of this first step could be supplemented with new insights. In addition, patients work on questions about the most burdening mental health problem and their own coping style (i.e. type of coping, how to mobilise individual resources). Relatives are asked to answer some of these questions from their perspectives. Finally, the patient starts with preparing their own treatment goals. - Equal to the first module, contacting peers is also possible while following the second module. - When the module is completed, patients and clinicians will also get a summary of the answered questions.

**Fig. 2:**
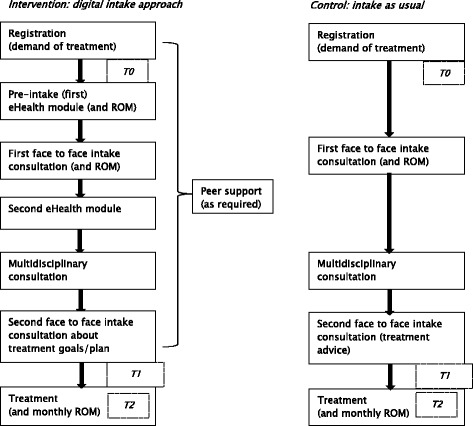
Digital intake process compared to intake as usual

In Fig. 2 the usual and new digital intake-processes are described. Patients of the intervention group follow the first pre-intake module before the first face to face intake consultation and the second module just after the first and before the second face to face intake consultation. Patients follow the eHealth modules at home on their own device, a computer or tablet. The estimated time for completing each module is 45 min. While filling out the eHealth modules, patients get the opportunity to have contact with peers (by phone or mail) who can support and prepare them for the intake consult. Peers have life experience with mental illness and treatment. They are already working at GGz Breburg and are experts in supporting patients. They have completed specific education about how to support patients using their experience in mental health care and get also instruction in the way to assist patients who are following eHealth. To prepare for the first consultation, the completed modules are visible for both the patient and the clinician. During this first intake consultation patients and clinicians discuss results of the first pre-intake module by exploring the burden and impact of the mental health problems on daily life. During the second face to face consultation patient and clinician complete, using the results of the second module, the exploration about problems, coping and goals and take shared decisions about the treatment policy and goals. After the second intake consultation the treatment starts. In both conditions the number of face to face contacts in the intake procedure are the same. To make sure that patients and clinicians have sufficient time to discuss the results of the eHealth module, in the new intake approach the first intake consult will take 30 min longer. Because ROM is a promising tool which visualises symptoms severity and functioning, stimulates effective communication between patients and clinicians and empowers patients [49, 53] in the new digital intake approach an additional ROM moment is planned. While the intake as usual includes a single ROM measurement linked to the face to face intake consultation, the new intake approach includes two measurements: one measurement incorporated in the first eHealth module and one, as usual, linked to the face to face intake consultation. In both conditions the multidisciplinary team consultation keeps a role in checking the quality and appropriateness of the proposed treatment and process steps, according to multidisciplinary guidelines.

To stimulate and facilitate the new way of working in the digital intake, the clinicians of the intervention teams follow a training of three half-day sessions in a period of up to four weeks. Aim of this training is gaining insight, knowledge and skills in the application of recovery supported care, shared decision making and eHealth with the purpose to facilitate patients to participate in their intake and treatment process and to stimulate an equivalent interplay between patients and clinicians. During the course of the study at least two follow-up training meetings are organised in the intervention group aimed to discuss clinicians’ experiences with the new way of working.

### Study flow

Patients who are referred to one of the participating departments, are invited for an intake consult. Patients who are planned for an intake consultation with a clinician of the intervention group, are assigned to the intervention team and will get the new eHealth intake automatically. Patients who have this first appointment with a clinician of the control group, are assigned to the control group and will follow the intake as usual. In addition, both groups of patients, who follow the new intake process and the intake as usual, will be invited to participate in the evaluation research consecutively. Research assistants inform all patients (who meet the inclusion criteria) about the research, ask them to participate in the study and obtain written informed consent.

Three measurement points will be scheduled (see Table 2): baseline assessment (T0) and two measurements (T1, T2) after the intake (T1 = two weeks after intake, T2 = two months after intake). Because only T1 and T2 contains additional questionnaires for this research, written informed consent will be asked during the first face to face appointment, just before T1. Patients who give informed consent for the research, are completing self-report questionnaires organised by independent research assistants. In addition to T1, clinicians answer questions about shared decision making regarding their patients. Patients and clinicians who participate in the study, receive a request by email to complete the questionnaires. If necessary, one week later they get a reminder by email. After nine and fourteen days the research assistant calls patients who have not yet completed the questionnaires. Patients who do not use internet, receive paper questionnaires by mail.

**Table 2:** Measurement points

Assessment/Questionnaire	Baseline (T0)	2 weeks after intake (T1)	2 months after intake (T2)
Primary Outcome Parameter			
Decisional Conflict			
Decisional Conflict Scale patient		X	X
VAS patient (additional)		X	X
VAS clinician (additional)		X	
Secondary Outcome Parameters			
Patient participation in mental health (treatment). Patient Participation Questionnaire		X	X
Shared Decision Making process SDM-Q-9 Patient and clinician.		X	X
Working alliance PDRQ-9		X	X
Adherence to treatment Drop-out, no-show		EPR	EPR
Symptoms-functioning SQ-48 (=ROM)	X	X	X
Patients’ characteristics			
Patients’ characteristics (sex, age, educational level, diagnosis, SQ-48 score, previous treatment at GGz Breburg, length of waiting)	EPD		
Motivation Demand for treatment on their own initiative? (1 item)	X		
Achieving personal treatment goals (1 item)			X

*EPR*, Electronical Patient Records

### Outcome measures

**Fig. 3:**
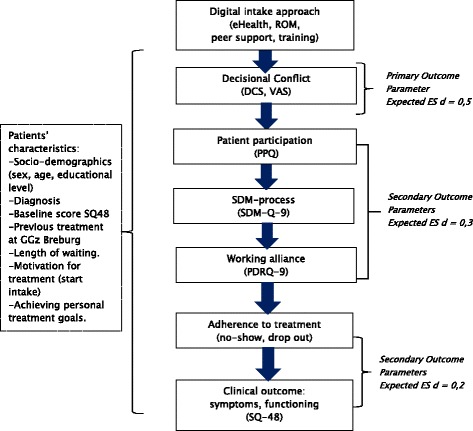
Primary and secondary outcome parameters in flow chart

The outcomes of interests will be decisional conflict (primary outcome), patient participation, shared decision making process, working alliance between patient and clinician, adherence to treatment and clinical outcome (secondary outcomes). The outcome measures are visualised in Fig. 3 and the measurement points in Table 2.

The level of decisional conflict is closely related to the secondary outcome parameters patient participation in treatment, shared decision making process and working alliance. Less decisional conflict is associated with more patient participation in decision making about treatment, a better shared decision making process and equality in the working alliance [26, 44, 54].

#### Patients’ characteristics

At baseline the following characteristics of patients are registered: socio-demographics (sex, age and educational level), diagnosis, SQ-48 (ROM) score, previous treatment, length of waiting time and the motivation to start treatment (own initiative or by pressure of the social environment). At T2, patients will receive a self-report questionnaire with a single question about the extent to which their personal treatment goals have been achieved. This question has three response categories (yes, partially, no).

$$ \mathrm{EPR}=\mathrm{Electronical}\ \mathrm{Patient}\ \mathrm{Records} $$

#### Primary outcome measure

The primary outcome measure is the degree of decisional conflict a central determinant of decision making, which is defined as ‘personal uncertainty about which option to choose’ [55, 56], will be measured with the translated Decisional Conflict Scale (DCS) [18, 55]. The DCS is a self-report questionnaire comprising sixteen questions about personal perceptions of: uncertainty in choosing options, modifiable factors contributing to uncertainty, effective decision making such as feeling the choice is informed, values-based, likely to be implemented and expressing satisfaction with the choice [55]. Each item is measured on a 5-point Likert scale (0 = strongly agree to 4 = strongly disagree). Besides the total score, the DCS has the following five subscales: uncertainty, informed, values clarity, support and effective decision. To calculate the total and subscale scores the item scores will be averaged and multiplied by 25. The scores thus range from 0 (no decisional conflict) to 100 (extremely high decisional conflict).

The psychometric properties of the English version of the DCS are adequate. Test-retest reliability (0.81) and Cronbach alpha (0.78) are high. The scale correlates to related constructs (knowledge, regret, discontinuance) and discriminates between groups who make and delay decisions (construct validity). DCS is responsive to change between different decision supporting interventions. The predictive validity of the DCS is also demonstrated [44, 55, 56].

To compare the degree of decisional conflict (or agreement) between patients and clinicians about the decisions made, at T1 both patients and clinicians fill out a Visual Analogue Scale (VAS).

#### Secondary outcome measures

The secondary outcome measures are patient participation, process of shared decision making, working alliance, patients’ adherence to treatment and clinical outcome.

The research team developed the Patient Participation Questionnaire (PPQ), because no accessible, brief instrument was found that measures patient participation in treatment with good psychometric properties and which is also suitable to fill out during or just after the intake process of mental health treatment.

The PPQ measures the role of the patient in treatment and shared decision making, consists of 14 items and is filled out by patients. Each item is measured on a five point Likert scale (1 = totally not applicable to 5 = totally applicable). Item scores will be summed. A higher score indicates a more active role of the patient in the interplay with the clinician and in mental health treatment. Three sample items of the PPQ include:

‘Along with my clinician I have described my treatment targets’.

‘I participate actively in my treatment’.

‘I participate in decisions about my treatment’.

After development, the PPQ was tested on nine clinicians and three peers working at GGz Breburg. They were asked to fill out the questionnaire and were interviewed afterwards for feedback. The aim of testing was to see if the items are relevant and comprehensible. This feedback resulted in textual improvements and adjustments in the order of the questions. The psychometric qualities of the questionnaire will be investigated in the population of this study: the factor structure, the reliability (test-retest and internal consistency) and construct (convergent) validity of the instrument [57, 58]. Convergent validity will be tested using

1. Decisional Conflict Scale (DCS) [55];
2. Visual Analogue Scale (VAS) consisting of one item filled out by patients, about the active role of the patient in treatment measured on a scale from 1 to 10 (1 = not at all to 10 = completely). A higher score means a more active role in mental health treatment;
3. two items about treatment targets of the Patient Reported Experience Measure (PREM) for chronical care [59, 60]. The items describe the joint formulation of treatment goals and how to achieve these goals. This self-report questionnaire is filled out by patients on a 5-point Likert scale (1 = completely disagree to 5 = completely agree);
4. Shared Decision Making-Questionnaire-9 (SDM-Q-9) [61–63] and
5. Patient-Doctor Relationship Questionnaire (PDRQ-9) [64].

The SDM-process will be measured with the Shared Decision Making-Questionnaire-9 (SDM-Q-9) [61, 62], which has Dutch versions for patients and clinicians [18, 63]. Both versions ask the patient and clinician first to enter the health problem the consultation was about and which decision was made. The questionnaire continues with nine items about the steps in the SDM process, scoring at a 6-point Likert scale that ranges from 0 (completely disagree) to 5 (completely agree). A total score can be calculated by summing the scores of all items. A high score indicates more SDM. The SDM-Q-9 shows a high reliability [61, 62]. The available results about the factorial validity are also positive [61, 62]. The psychometric testing of the Dutch version of the SDM-Q-9 [63] demonstrated good acceptance, internal consistency, and acceptable to good convergent validity of the versions for patients and physicians. Patients will be asked to complete the SDM-Q-9 at T1 and T2. At T1 clinicians will complete the same questions regarding the consultations with their patients.

The PDRQ-9 will measure the working alliance. The PDRQ-9 consists of 9 items and measures the therapeutic aspects of the clinician-patient relationship from a patient point of view. The items focus on the empathic style and availability of the clinician and are answered on a 5-point Likert scale (1 = not at all appropriate to 5 = totally appropriate). Two sample items are: ‘My clinician understands me’ and ‘My clinician and I agree on the nature of my medical symptoms’. A mean score of all nine items is calculated. A higher score means more satisfaction about the relationship. The psychometric quality of the Dutch PDRQ-9 shows good internal consistency, adequate test-retest reliability and the ability to discriminate between patient groups [64].

Next the patients’ adherence to treatment will be investigated. Adherence can be defined as the extent to which the patient’s behaviour concur with the advice of the clinician [9]. In this study adherence is operationalised as the number of missed appointments (no-shows) and patients who do not want to proceed with treatment (early treatment drop out). These data will be extracted from the electronic patient records.

Finally, effects on treatment results will be studied with the Symptom Questionnaire-48 (SQ-48). This is a self-report questionnaire for the measurement of psychological distress, vitality and work functioning and consists of 48 items. Each item is rated on a 5-points Likert-scale (0 = Never to 4 = Very often). Five subscales cover aspects of psychopathology: Depression, Anxiety, Somatization/Somatic complaints, Social Phobia and Agoraphobia. In addition, four subscales were constructed to assess specific aspects of behaviour and/or functioning: Aggression, Cognitive problems/complaints, Work and Vitality. The total score is calculated by summing 37 items (excluding work and vitality subscale-items) and ranges from 0 to 148, with higher scores indicating more psychological distress. The subscales work and vitality have a different scoring (Work with answering options “not applicable”; Vitality which scoring is reversed for the positively formulated items).

Research demonstrated the quality of the psychometric properties of the SQ-48. The internal consistency (reliability) as well as the convergent and divergent validity among both clinical and nonclinical samples are good [65]. Research also showed that the SQ-48 has excellent test-retest reliability and good responsiveness to therapeutic change [66].

#### Treatment integrity

To check the treatment integrity, during the implementation and period of data collection, process indicators will be assessed. These indicators will report the degree of completion of the eHealth modules (registered by research assistants in the research dbase), number of completed ROM measurements (data extraction from electronic patient records) and the extent of using peer support (registered by peers themselves).

#### Sample size calculation

This study is designed to detect a medium effect size of *d* = 0.5 on the primary outcome parameter between the intervention and control group. A significance level set at α = 0.05 and 65 patients per arm will yield a power of 0.80 [67]. Due to the cluster-randomisation at team level, we will calculate the effective sample size with an intra cluster correlation coefficient (ICC). The ICC is a measure of relatedness of responses within a cluster [68]. When adjusting for clustering within the matched pair teams of each department we expect an ICC = 0.01. Using the following formula [68]: Design Effect (DE) = 1 + (m-1) ICC (0.01) (m = number of subjects in a cluster), the sample size needs to be 77 patients per arm. We expect about 10% of the participants to drop out of the study. This requires an initial inclusion of 88 patients per arm, which means that in total 176 patients and on average 44 patients per centre (22 patients per intake-team per arm) will need to be included.

#### Statistical analyses

The data will be analysed according to the intention-to-treat principle. In addition, a completer case analysis will be performed.

Descriptive analysis will be conducted to describe patients’ characteristics (age, gender, educational level, diagnosis, SQ-48 (ROM) score at T0, motivation for treatment at T0, previous treatment and length of waiting) to check the similarity between the intervention and control group. In addition, treatment integrity (degree of completion of the eHealth modules, number of completed ROM measurements and the extent of using peer support) will be described.

Due to the cluster randomisation, the hypotheses will be tested using multi-level analysis [45], which is also a flexible statistical method in handling missing data [69]. We assume a three level structure: clusters (departments), patients and multiple measurements over time (within patients) [70].

The patients’ and clinicians’ views on the Shared Decision Making process (SDM-Q-9) and Decisional Conflict (VAS) will be compared using independent t-tests.

Analyses will be conducted using SPSS 19.0 and MLwiN 2.21. Reporting of the results of the study will be in accordance with the CONSORT statement 2010 (extension cluster randomised trials).

## Discussion

The described study is designed to test, in a two-arm cluster-randomised controlled trial, the efficacy of a digital exploration of patients’ treatment needs and preferences preparing patients and clinicians for the intake consultations facilitated by Routine Outcome Monitoring (ROM), peer support and training of clinicians, in terms of the primary outcome the degree of decisional conflict about choices in treatment. Secondary outcomes of the study focus on patient participation, shared decision making process, working alliance, adherence to treatment and clinical outcomes.

Currently, patients in specialised Dutch mental health care usually have a dependent, inactive role in the intake and treatment process with a more dominant, active role of the clinician [28–31]. This intervention offers the opportunity for patients to participate actively in their mental health treatment with an equivalent interplay with their clinician, which is an important base for shared decision making aiming to reduce decisional conflict. Moreover, the new digital intake approach may improve patients’ adherence to treatment and clinical outcomes. As previously explained, the intervention responds to the advantages of shared decision making [19–29], the promising benefits of patient participation in mental health treatment [4–9] and the importance of improving the quality and equivalence of the working alliance [9, 40, 42, 43]. Hence, this initiative is focused on changing clients’ and clinicians’ roles, at both sides of the dyad, from the start of treatment.

A strength of this study is the reduced risk of confounding by the matched pair design. Randomisation is conducted at cluster level within each department between two similar teams of intake-clinicians (matched pairs). Helping to prevent cross-over effects, the intervention and control teams of intake-clinicians participate in different multidisciplinary team consultations.

A second strength is that the additional data collection for this research is carried out independent of the treatment. Separately for this study patients fill out questions about decisional conflict, participation in mental health treatment, shared decision making and the working alliance with the clinician. This results are not visible at patient level during intake and treatment. Data collection is conducted by independent research assistants. This approach diminishes undesired influence of the research team or clinicians on the results, reduces the chance of social desirable answers and enhances uniformity and quality of the collected data. Only the outcome parameters no-show, drop-out and clinical outcome (ROM) will be collected in the context of treatment.

The third strength of the study is that at T1, both the patients and clinicians are invited to complete a questionnaire about decisional conflict and shared decision making. If there are different views between patients and clinicians about the application of shared decision making, they will be detected and further analysed.

Finally, the study is conducted in four departments working in two regions and treating different diagnostic groups (depression, anxiety and personality disorders). Because the data collection takes place in real world clinical practice and in several departments the results will be generalizable to a broader group of mental health care teams, clinicians and patients with depression, anxiety and personality disorders.

The study brings about some limitations that could influence the results. First, the clinicians are not blinded for the design. It is possible that clinicians, working in the control teams, make additional efforts to stimulate patient participation, to improve the quality of the working alliance and to applicate shared decision making. Although, the research assistants are partially blinded for the study arm, they are less likely to influence the results, because the outcome parameters are measured by self report questionnaires, filled out by patients and clinicians. Furthermore, it is not possible to prevent contamination bias completely. There is a chance that knowledge about and experiences in the new way of working will cross over from clinicians of the intervention group to the intake-clinicians of the control group. Cross over effects between patients are unlikely, because patients of the intervention and control group follow individual treatment sessions and hence do not meet and know each other. Besides, probably there will be personnel changes in the participating teams which may affect the study and its findings. If this is the case the investigator will deliberate with the manager of the department about the solution and monitors closely that no switches between intervention and control group will take place, replacement of a clinician is permitted by the same discipline and new clinicians in the intervention group will be trained. Finally, to discuss the results of the intake module, in the intervention group the initial intake consult takes thirty minutes extra time compared to the control group. This extra time with the clinician could be a confounder, because it may enable patients to become more actively involved in the dialogue about their own treatment.

The findings from this study will provide valuable information with regard to the efficacy of a digital intake approach facilitated by ROM, peer support and training of clinicians. The study will answer the question whether this intervention helps to reduce decisional conflict and enhance patient participation, shared decision making, working alliance, adherence to treatment and clinical outcomes. The findings of this study may give an evidence base for future eHealth initiatives in the intake process, which may contribute to the roll out of such interventions aiming to improve the participation of patients in decision making about their mental health treatment.

## Abbreviations

SDM: Shared decision making
ROM: Routine outcome monitoring
